# The effects of Olmesartan/amlodipine administered in the Morning or At Night on nocturnal blood pressure reduction in Chinese patients with mild-moderate essential hypertension (OMAN Trial): study protocol for a prospective, multicenter, randomized, open-label clinical trial {1}

**DOI:** 10.1186/s13063-023-07726-x

**Published:** 2023-11-28

**Authors:** Mengzhuo Xu, Xin Zhang, Runyu Ye, Xueting Liu, Lirong Sun, Shanshan Jia, Zhipeng Zhang, Xinran Li, Ziqiong Wang, Hang Liao, Rufeng Shi, Kai Liu, Si Wang, Qingtao Meng, Xiaoping Chen

**Affiliations:** Cardiology Department, West China Hospital, Sichuan University, Chengdu, Sichuan Province 610041 People’s Republic of China

## Abstract

**Introduction:**

Hypertension increases the risk of cardiovascular disease. Uncontrolled nocturnal blood pressure is prevalent in patients taking antihypertensive medication, with an incidence rate of 30–60%. Although chronotherapy with antihypertensive agents may provide a new direction for effective control of nocturnal blood pressure, the clinical evidence base remains controversial. This research is presently underway to compare the effects of morning and bedtime administration of antihypertensive medication on nocturnal reduction and circadian rhythm of blood pressure in patients with hypertension.

**Methods and analysis:**

This study is being performed as a randomized, multicenter, open-label, parallel-group, clinical trial in which 720 participants are to undergo 24-h ambulatory blood pressure measurement (ABPM) and office blood pressure measurement (OBPM) at baseline before being randomly assigned to a morning (6–10 am) or a bedtime (6–10 pm) administration group. Each participant receives one 20/5-mg tablet of olmesartan/amlodipine (OA) daily for 4 weeks and is then followed up at 4-week intervals for a total of 12 weeks. During follow-up, the OA dosage is adjusted according to the ABPM and OBPM results. Patients with uncontrolled hypertension at the first follow-up visit will receive an increase in OA dosage to 1.5 tablets/day. For patients with blood pressure that is still uncontrolled after a further 4 weeks, the dosage of OA can be increased to 2 tablets/day. The primary objective is the reduction in mean nocturnal systolic blood pressure between baseline and week 12. The secondary objectives are the reduction in ambulatory blood pressure at weeks 4 and 12 and the blood pressure control rate at weeks 4, 8, and 12.

**Discussion:**

Antihypertensive chronotherapy remains controversial. A superiority test hypothesis design has been adopted for this trial, in which all participants will be taking the same antihypertensive medication. We anticipate that our findings will determine if nocturnal blood pressure control in Chinese patients with essential hypertension varies according to whether antihypertensive medication is taken in the morning or at bedtime. This study may provide scientific evidence for the application of chronotherapy in clinical practice.

**Trial registration:**

ChiCTR2200059719. Registered on 10 May 2022

(http://www.chictr.org.cn/edit.aspx?pid=169782&htm=4) {2a,2b}

**Supplementary Information:**

The online version contains supplementary material available at 10.1186/s13063-023-07726-x.

## Introduction {6a}

Hypertension is the most common chronic non-communicable disease and the most important risk factor for cardiovascular disease worldwide. Moreover, hypertension significantly increases the risks of atherosclerotic heart disease, stroke, chronic kidney disease, and heart failure. According to the recent China Hypertension Survey, the age-adjusted prevalence of hypertension in Chinese adults aged >18 years is 23.2%, with the estimated number of affected patients exceeding 250 million; however, the blood pressure (BP) control rate is only 16.8%, which is significantly lower than that in the developed countries of Europe and the USA [1]. In 2017 alone, 2.54 million Chinese individuals died as a result of high systolic BP (SBP), with cardiovascular disease being the immediate cause of death in 95.7% of cases [2]. Therefore, effective control of BP is critical in the prevention and treatment of cardiovascular disease and for the reduction of its high mortality rate.

The Patient-Centered Cardiac Event Evaluation Million Population Project, which included 1.7 million community-dwelling residents aged 35–75 years in 31 provinces and autonomous regions in mainland China, found that 81.1% of treated hypertensive patients took a single antihypertensive agent but that the proportion of patients with uncontrolled BP on combination therapy was only 18.6% [3]. This finding suggests that low utilization of combination therapy may be an important reason for the low BP control rate in hypertensive patients in China. The single-pill combination (SPC) is an oral antihypertensive regimen that includes two or more agents. Several clinical trials have demonstrated that SPC is safe and effective in protecting target organs, reducing/offsetting adverse reactions, increasing BP control synergistically, and lowering the incidence of cardiovascular disease and deaths from the disease [4]. Therefore, SPC is recommended as the initial choice in the recently published international hypertension guideline [5]. Olmesartan/amlodipine (OA) is a novel SPC formed by the combination of an angiotensin receptor antagonist and a calcium ion blocker and may effectively lower peripheral vascular resistance and have an antihypertensive effect without affecting glucose or lipid metabolism. Previous large-scale clinical studies have confirmed that OA can significantly reduce BP in hypertensive patients who do not meet their BP target on monotherapy [6, 7]. However, those studies used only office BP as the indicator of efficacy. Therefore, there is a lack of information on the effectiveness of OA in reducing 24-h BP, daytime BP, nighttime BP, and morning BP as well as in restoring the circadian rhythm of BP.

According to recent studies, uncontrolled nocturnal BP is particularly common in hypertensive patients on antihypertensive medication [8], with an incidence rate of 30–60% depending on the study cohort [9–12]. Mean nighttime BP is a better predictor of cardiovascular events than daytime BP or 24-h mean BP [13]. A prospective cohort study demonstrated that every 20 mmHg increase in nighttime BP was associated with a 23% increase in the risk of all-cause mortality and a 36% increase in cardiovascular and cerebrovascular events [14]. Controlling nighttime BP and restoring the circadian rhythm of BP are effective ways of reducing the risk of cardiovascular and cerebrovascular events in hypertensive patients. However, managing nocturnal BP is challenging in practice. Antihypertensive chronotherapy may provide a new direction for effective management of nocturnal BP. A recently published multicenter, prospective, randomized controlled study from Spain (known as Hygia Chronotherapy Trial) found that taking antihypertensive medication before bedtime significantly reduced the risk of cardiovascular death, myocardial infarction, and major cardiovascular composite events (including coronary revascularization, heart failure, or stroke) in hypertensive patients [15]. However, a two-center randomized, crossover, prospective clinical study performed in London and Thessaloniki suggested that the timing of antihypertensive drug delivery (morning vs. nighttime) did not affect 24-h BP, daytime BP, or nocturnal BP levels in hypertensive patients [16]. Furthermore, the clinical evidence for antihypertensive chronotherapy comes mostly from populations on a single antihypertensive agent in Europe or the USA or from populations with cardiovascular disease. High heterogeneity, including in intervention methods, evaluation indicators, and study populations, between these studies has been noted [17]. Moreover, a systematic review of 21 studies that compared antihypertensive medication taken in the morning with that taken at bedtime indicated that both dosing strategies showed no statistical difference in 24-h mean BP reduction values [18]. Thus, the clinical benefit of taking antihypertensive drugs before bedtime remains controversial.

Therefore, we have initiated this prospective, randomized, open-label, parallel-group clinical study to compare the effects of morning and bedtime administration on nocturnal BP reduction and recovery of circadian rhythm in patients with essential hypertension. The results of this trial are expected to provide useful guidance for optimizing BP control, particularly in Chinese patients with hypertension.

## Study objectives {7}

### Primary objectives

The primary objective will be the decrease in average nocturnal SBP between baseline and after 12 weeks of the intervention. Average nocturnal BP is defined as the mean BP reading during the period between falling asleep and waking up [19] and baseline nocturnal BP is defined as the average nocturnal BP recording at baseline (one without taking any antihypertensive medication or after 2 weeks off medication).

### Secondary objectives

#### 24-h ambulatory BP measurements

1. Reductions in 24-h and daytime average SBP and diastolic BP (DBP) at weeks 4 and 12.
2. Average 24-h, daytime, and nocturnal SBP and DBP control rates at weeks 4 and 12.
3. Proportion of non-dippers, the nocturnal BP reduction rate, and coefficient of variation for BP at weeks 4 and 12.
4. Morning surge of BP (MSBP): two widely accepted definitions of MSBP will be quantified, namely, the pre-awakening morning surge and the sleep-trough morning surge. The pre-awakening morning surge will be determined by considering the difference between the average BP recordings in the 2 h after rising from sleep and those over 2 h before rising from sleep. The sleep-trough morning surge will be determined as the difference between the average BP over 2 h after rising from sleep and the average of three consecutive BP recordings centered on the lowest reading during sleep. The MSBP will be measured at weeks 4 and 12.

#### Office BP measurements

1. The reduction in average SBP and DBP in the clinic at weeks 4, 8, and 12.
2. Office BP control rates at weeks 4, 8, and 12.
3. The treatment response rate (SBP <140 mmHg or a decrease ≥20 mmHg and DBP <90 mmHg or a decrease ≥10 mmHg) at weeks 4, 8, and 12.

#### Proportion of patients requiring intensive treatment

The patients requiring intensive treatment in week 4, which includes masked uncontrolled hypertension (office BP <140/90 mmHg and 24 h ambulatory BP ≥130/80 mmHg, daytime ambulatory BP ≥135/85 mmHg, or nighttime ambulatory BP ≥120/70 mmHg) and sustained uncontrolled hypertension (office BP ≥140/90 mmHg, 24 h ambulatory BP ≥130/80 mmHg, daytime ambulatory BP ≥135/85 mmHg, or nighttime ambulatory BP ≥120/70 mmHg). In addition, the patients who do not meet the office BP target (office BP <140/90 mmHg) at week 8 also need intensive treatment. Then, the proportion of patients requiring intensive treatment at different follow-up visits will be calculated.

#### Safety evaluation {22}

The safety evaluation will include potential drug side effects (including hypotension, dizziness, coughing, and ankle edema), the incidence of severe adverse events, and the proportion of extreme dippers.

## Methods and analysis

### Design and study population {8, 9}

This prospective, multicenter, randomized, open-label, parallel-group clinical trial is being performed at 20 hospitals across Sichuan in China. The framework of the trial is superiority. We used the SPIRIT reporting guidelines to standardize our study (Additional file 1). The study started in June 2022 and is scheduled to conclude in September 2023. Chinese patients aged 18–75 years with a diagnosis of essential hypertension are eligible to participate. Table 1 shows the study inclusion and exclusion criteria.

**Table 1:** Inclusion and exclusion criteria for patient recruitment {10}

**Inclusion criteria**	**Exclusion criteria**
1. Patients with essential hypertension, aged 18–75 years, of either sex, with regularly scheduled activities and work and rest durations 2. Patients who have not previously received antihypertensive treatment or stopped using antihypertensive agents for 2 weeks before starting the trial medication 3. Able to provide a signed informed consent form and willingness to attend for follow-up in a timely manner	1. Extreme dipper (nocturnal BP >20% lower than daytime BP) 2. Pregnancy, planning a pregnancy, or breastfeeding 3. Renal artery stenosis 4. Hyperkalemia (serum potassium >5.5 mmol/L), chronic renal insufficiency (creatinine >265 μmol/L) 5. History of or progressing toward malignant hypertension, hypertensive emergency, hypertensive crisis, or hypertensive encephalopathy during the 2-week antihypertensive drug withdrawal phase and enrollment 6. History of drug use or other causes of angioedema or a history of hypersensitivity to angiotensin receptor antagonists, angiotensin-converting enzyme inhibitors, or renin inhibitors 7. History of alcohol or drug abuse 8. Working at night or shift work 9. Cardiovascular disease, such as unstable angina, heart failure, life-threatening arrhythmias, atrial fibrillation, renal failure, hypertrophic cardiomyopathy, and grade III–IV retinopathy 10. Unable to tolerate ambulatory BP measurement or participate in clinical research 11. History of allergy to amlodipine 12. History of hemorrhagic stroke 13. Participating in or planning to participate in other trials 14. Poor compliance

### Sample size calculation {14}

Considering the lack of published studies on the nocturnal systolic BP reduction after treatment with a fixed-dose OA combination, we referred to the study on BP control reported by Wang et al. [20], in which nocturnal systolic BP was reduced by −14.7 ± 11.6 mmHg in patients who received a fixed-dose combination of valsartan/amlodipine. We also collected and analyzed the medical data for 50 patients who had received OA at our center. Our preliminary analysis revealed that the standard deviation (SD) for nocturnal SBP reduction was 12 mmHg in subjects who took OA for 3 months. According to the above analysis, we set SD as 12 when calculating the sample size. In terms of the endpoint, a superiority margin of 3 mmHg was considered a clinically meaningful BP reduction on the basis of the observed decreases in cardiovascular morbidity with small reductions in systolic BP (2–5 mmHg) by pharmacological therapy. After discussion with clinical experts, we selected 3 mmHg as the superiority margin. Hence, while the significance level was set to 0.05 (two-sided) and the power to 85%, 289/289 cases would be needed for each category. Assuming a 20% dropout rate, each study group would comprise at least 360 patients, for a total of 720 cases. PASS version 15.0 was used to calculate the sample size.

### Patient and public involvement

No patient was involved in the protocol design.

### Randomization strategy {15, 16a, 16b, 16c}

This study will enroll 720 patients from 20 hospitals across Sichuan in China. We used the *Research Randomizer* (available at www.randomizer.org) to generate random numbers from 1 to 36 per set, for a total of 20 sets representing various research units. The random number in each set is filled in the 36-row table that alternates between morning and bedtime administration groups. Based on the random numbers we set, the participants from each unit are randomly assigned to the morning or bedtime administration group according to their order of recruitment. Specialized staff from the main research unit are responsible for generating the allocation sequence and are not involved in the interpretation of the ambulatory BP report or patient recruitment.

### Interventions {6b, 11b, 11d, 13}

**Fig. 1:**
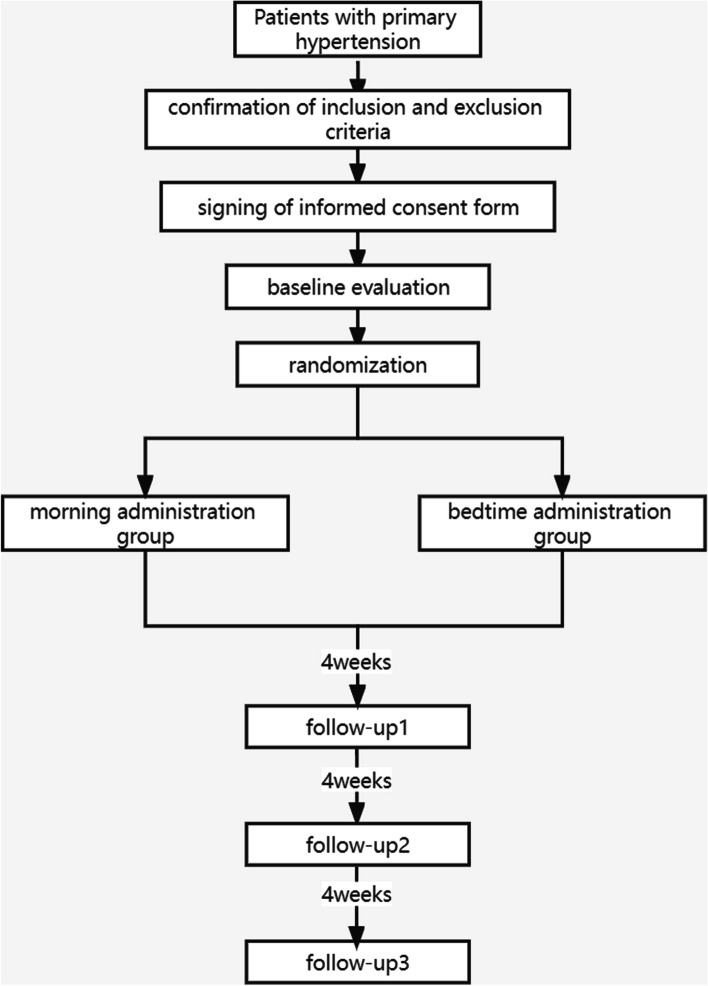
The diagram of this trial

Study participants are randomized in a 1:1 ratio to a morning (6–10 am) or a bedtime (6–10 pm) administration group. Initially, each participant receives one tablet of OA (20/5 mg) daily for 4 weeks and is then followed up at 4-week intervals for a total of 12 weeks. The OA dosage is adjusted according to the results of ABPM and OBPM during follow-up. Controlled BP is defined as a 24-h mean BP <130/80 mmHg, daytime mean BP <135/85 mmHg, nighttime mean BP <120/70 mmHg, and OBPM <140/90 mmHg. The OA dosage is increased to 1.5 tablets/day in patients who are found to have masked or sustained uncontrolled hypertension at the initial follow-up visit. If BP remains uncontrolled after a further 4 weeks, the OA dosage can be increased further to 2 tablets/day. All participants are instructed to discontinue their preexisting antihypertensive medications for at least 2 weeks before starting the trial intervention. Adherence with medication is recorded at each follow-up visit. Concomitant use of other antihypertensive drugs other than OA is prohibited during this study. Figure 1 illustrates the current study’s diagram.

### Measurement of outcomes {12}

#### 24-h BP measurement

ABPM readings are obtained from the non-dominant arm at baseline and weeks 4 and 12 using a TM2430 BP monitor (A&D Inc., Tokyo, Japan). During monitoring, BP is measured at 20 min intervals throughout the day (usually from 6 am to 10 pm) and then at 30 min intervals at night (usually from 10 pm to 6 am). Daytime is defined as the clock time interval from 8am to 6 pm, nighttime as the clock time interval from 11 pm to 5 am, and morning as the clock time interval from 5 am to 8 am. Valid recordings will span >20 h, including at least 20 during the day and 10 during the night [21]. A diary card is issued to each participant so that they can record their schedule in detail, including the times they go to bed and wake up. Mean 24 h BP is calculated as the average of all successful readings. Nocturnal BP is defined as the mean BP during the interval between falling asleep and waking up. Daytime BP is defined as the mean value of all other readings. The researchers obtain the average 24 h, daytime, and nocturnal BP values by analyzing the original data from the telemedicine platform.

#### Office BP measurement

Office BP will be measured using calibrated electronic sphygmomanometers (HBP-9020, Omron Corp., Kyoto, Japan). To obtain accurate BP data, all participants are requested to rest for 5 min before the measurement, and the best measurement time is assigned between 8 am and 10 am. SBP and DBP are obtained three times on the right arm in a sitting position. The average of the three readings will be used in the final analysis.

#### Collection of medical data and quality control {5d, 11a, 11c, 18a, 18b, 19, 21a, 21b, 23, 27}

This research was initiated by Professor Xiaoping Chen at the Hypertension Center, West China Hospital, Sichuan University. Trained physicians, nurses, and clinical research coordinators (CRCs) are required to keep medical records and collect data in accordance with the study protocol. The medical officer assigned to each study participant is alerted to remind the participant to attend all interventions and assessments in an effort to minimize the dropout rate. Participants and their caregivers also have direct access to a hotline during office hours if they have any concerns or problems. All original research data are entered into the electronic data capture system, namely, the Red Shine Chronic Disease Management System developed by the Hypertension Center at West China Hospital. All study-related information is stored securely in the system and all information concerning individual study participants is stored in locked filing cabinets in areas with limited access. A training session on the study protocol was provided before initiation of the study at each participating site to ensure that the quality of this clinical trial is satisfactory. On-site monitoring at the beginning, middle, and the end of the study is performed by assigned clinical researchers to ensure complete compliance with the study protocol. A project specialist has been assigned to this trial for data monitoring, examination, and cleaning. This specialist will perform the interim analyses, the frequency of which will be decided by the specialist in consultation with the principal investigator. The specialist will advise the principal investigator of the interim findings if necessary. After data cleaning is completed, the data managers and statisticians will conduct a final review of unresolved data issues, discuss the division of datasets together according to the statistical analysis plan, and examine any reports of serious adverse events. A statistical analysis is to be conducted halfway through and at the end of the trial. The subjects will be discontinued from follow-up if individual participants report any serious adverse effects during the intervention or a significant deterioration in their mental or physical state. This decision would be made by the principal investigator. Table 2 presents an overview of the measurement methods and data collection for the entire study cohort.

**Table 2:** Data collection in this trial variables

Variable		Baseline	Week 4	Week 8	Week 12
Demographics	Name, age, sex, and race	×			
Medical history collection	Past medical history	×			
Physical examination	Height, weight, and waist circumference	×	×	×	×
	Heart rate	×	×	×	×
	Breath rate	×	×	×	×
	Office BP measurement	×	×	×	×
Laboratory examination	Blood routine examination	×			
	Blood biochemistry	×			
	Urine routine examination	×			
	Urine microalbumin	×			
ABPM	24-hour BP measurement	×	×		×
Compliance with medication	BMQ	×	×	×	×
Level of health literacy	CHLSH scale	×			
Concomitant medications	—	×	×	×	×
Adverse event record	—	×	×	×	×

*BP* Blood pressure, *ABPM* Ambulatory blood pressure monitoring, *CHLSH* Chinese Health Literacy Scale, *BMQ* Brief Medication Questionnaire

## Statistical analyses {20a, 20b}

### Descriptive analysis

The study population is divided into a morning administration group and a bedtime administration group. Quantitative variables will be summarized as the mean ± SD or as the median [interquartile range (IQR)] depending on the type of the original data. Qualitative variables will be presented as the absolute and relative frequency. Quantitative variables will be compared between the two study groups using the* t*-test or Mann–Whitney *U* test and qualitative variables using the chi-squared test or Fisher’s exact test.

### Analysis of primary and secondary objectives {12}

All analyses will be based on the intention-to-treat (ITT) and the per-protocol (PP) populations. The primary objective of the study will be analyzed using an analysis of covariance (ANCOVA) model with appropriate baseline adjustments using the following covariates: baseline BP, age, sex, body mass index, alcohol consumption status, smoking status, fasting glucose, total cholesterol, low-density lipoprotein cholesterol, and the course of hypertension. Only covariates for which the results are statistically significant will be selected as explanatory variables for inclusion in the final model. The ANCOVA model will also be used to evaluate the other secondary objectives. Mean differences or odds ratios will be determined across groups along with their 95% confidence intervals. In the final analysis, we also aim to determine the antihypertensive dosage titration ratio for the two groups and then separately analyze the BP reduction further in subgroups whether or not the antihypertensive dosage was titrated. All statistical analyses will be performed using SPSS version 26.0 (IBM Corp., Armonk, NY, USA). The significance level will be set to 5% for all statistical tests.

### Handling of missing data {20c}

The process for handling missing data is generally divided into the deletion of cases with missing data and imputation of missing data. The commonly used methods include mean imputation, last observation carried forward, and multiple imputation. We will deal with the missing data depending on the circumstances under the guidance of the Department of Clinical Research Management at West China Hospital.

## Discussion

Thus far, neither the European Society of Cardiology nor the American Heart Association has established guidelines regarding the best time of day for patients to take their antihypertensive medication. Therefore, antihypertensive chronotherapy is a hot topic in the hypertension field across the world. The Hygia Chronotherapy Trial [15] reported that cardiovascular event rates would be significantly reduced in subjects who took more than one antihypertensive agent at bedtime. Subsequently, the results of the Treatment In Morning versus Evening (TIME) study [22], which was designed to answer the question of when patients should take antihypertensive medication for the most favorable cardiovascular outcomes, were presented at the 2022 European Society of Cardiology Annual Conference. Unlike the Hygia Chronotherapy Trial, the TIME study strictly limited the time of day when oral antihypertensive medication was taken in each of the randomized groups and found no significant difference in the cardiovascular event rate between the morning and evening medication groups. The TIME investigators suggested that patients with essential hypertension could take their medication in the morning or evening based on their habits or simply for convenience. However, the TIME study did not assess nighttime BP and did not answer the question of whether morning or evening medication interventions in non-dippers or reverse-dippers affected cardiovascular outcomes, which warrants further exploration. In our study, 24-h ABPM is being performed at baseline and after 4 and 12 weeks of treatment, which can be supplemented with supporting data to the difference of nocturnal SBP reduction between morning and bedtime medication.

Although OA is a new oral SPC with a relatively long half-time, the findings of this study may not be generalizable to other antihypertensive SPCs. Olmesartan was found to antagonize the vasoconstrictor and pressor responses induced with angiotensin II in a dose-dependent manner in animal studies, with an elimination half-life of 10-16h, it allows once-daily administration [23]. However, pharmacokinetic and pharmaco-dynamic differences have been demonstrated between a fasting group and a group fed a high-fat meal [24], which indicates that dietary factors may have a significant effect on the absorption of olmesartan. Furthermore, amlodipine has a linear pharmacokinetic profile and shows a strong positive correlation between the oral dose administered and the peak plasma concentration and area under the plasma concentration-time curve. Amlodipine undergoes extensive but slow hepatic metabolism [25] and has a longer half-life of approximately 30–50h. Accordingly, our ongoing trial should provide specific information on the antihypertensive effectiveness of OA when taken at daytime and at nighttime rather than general information for all types of antihypertensive agents.

## Limitation

This study will have some limitations. First, it only includes Chinese individuals. Therefore, our findings may not be applicable to other ethnic populations. Second, ABPM is being performed over 24 h, which may not be as accurate as 48 h of monitoring. Third, one will exhibit a different BP response to a similar dose administered at bedtime or in the morning. Therefore, a randomized crossover trial will be required to examine the antihypertensive effectiveness of morning versus bedtime dosing. Fourth, patients in this trial are being followed for 3 months only, so it will not be possible to assess cardiovascular outcomes in the longer term.

## Trial status {3}

This trial is ongoing. This protocol is the third version and was revised on March 2022. Recruitment commenced in June 2022 and follow-up of the last patient recruited is estimated to be completed in September 2023.

## Conclusion

This prospective, multicenter, randomized controlled clinical study of antihypertensive chronotherapy in Asian patients aims to bridge the present knowledge gap in the field of antihypertensive chronotherapy and to provide clinical evidence on its use in hypertensive patients in China. Based on our research, we hypothesize that taking antihypertensive medication before bedtime can improve both nocturnal BP and the circadian rhythm of BP, thereby minimizing the risk of cardiovascular events.

## Dissemination plans {31a}

The results of this study will be actively disseminated through scientific publications and conference presentations.

## Provisions for post-trial care {30}

All participants will continue to be followed up in hypertension outpatient clinics. We would also provide compensation in the event of an unforeseen accident.

### Supplementary Information

**Additional file 1.** Reporting checklist for protocol of a clinical trial.

## Data Availability

Data will be available upon request to the investigator.
